# Complex reconstructions in head and neck cancer surgery: decision making

**DOI:** 10.1186/1758-3284-3-14

**Published:** 2011-03-08

**Authors:** Imke C Wehage, Hisham Fansa

**Affiliations:** 1 Department of Plastic, Reconstructive and Aesthetic Surgery, Hand Surgery, Bielefeld, Germany

## Abstract

Defects in head and neck after tumor resection often provide significant functional and cosmetic deformity. The challenge for reconstruction is not only the aesthetic result, but the functional repair. Cancer may involve composite elements and the in sano resection may lead to an extensive tissue defect. No prospective randomized controlled studies for comparison of different free flaps are available. There are many options to cover defects and restore function in the head and neck area, however we conclude from experience that nearly all defects in head and neck can be closed by 5 different free flaps: radial forearm flap, free fibula flap, anterior lateral thigh flap, lateral arm flap and parascapular flap.

## Introduction

Resection of advanced-staged malignancies of the upper aerodigestive tract often require advanced reconstructive procedures. Many patients suffer from poor nutrition, pulmonary disease, arteriosclerosis, liver insufficiency and clotting disorders.

The ideal reconstruction should be performed in a single stage operation, should be reliable and restore swallowing. Besides direct closure, healing by secondary intention, skin grafts and local flaps, free flaps should be performed depending on the patient's needs. Limited life expectancy in tumor patients argues for a reconstructive method that will return the patient to reasonable function in a time appropriate to the history of the tumor.

### Local pedicled flaps

The **deltopectoral flap **came up in the 70ies, first described in 1971 by Bakamjian [1,2]. The reconstruction can be performed in a single-stage operation, but a second operation is required to divide the pedicle after the skin island has regained its vascularisation from local tissue. The pedicle for this flap arises from the internal mammary artery, the application for intraoral or pharyngeal reconstruction is limited because of the pedicle length.

The pectoralis maior musculocutaneous flap is sourced by the pectoral branch of the acromiothoracic branch and became a workhorse for head and neck reconstruction. Aryian was first to describe this flap in 1979 [3]. The limitation of the flap is the bulkiness-especially in female patients. The pedicle length reaches up to 7 cm.

The pedicled latissimus dorsi musculocutaneous flap was first described to cover defects in head and neck in 1978 by Quillen [4], the flap came up in 1896 (Tansini) [5]. As a free flap the latissimus was first described in 1979 by Watson [6]. Based on the thoracadorsal artery the harvesting of the flap requires an intraoperative change of the patient's positioning, the flap is bulky and implies relatively high donor site morbidity such as seroma. To avoid bulkiness, the flap can be harvested as a muscle flap covered with skin graft. A perforator flap with the same pedicle excluding the muscle was described by Cavadas in 2002: the TAP flap (thoracodorsal artery perforator flap) [7].

The internal mammary artery perforator flap (IMAP) is a new flap, which can be harvested from the ventral thorax either horizontally or vertically orientated. Rotation arc allows defect closure at the neck and lower head. However, its application has to be defined yet [8].

### Free microvascular flaps

**Figure 1:**
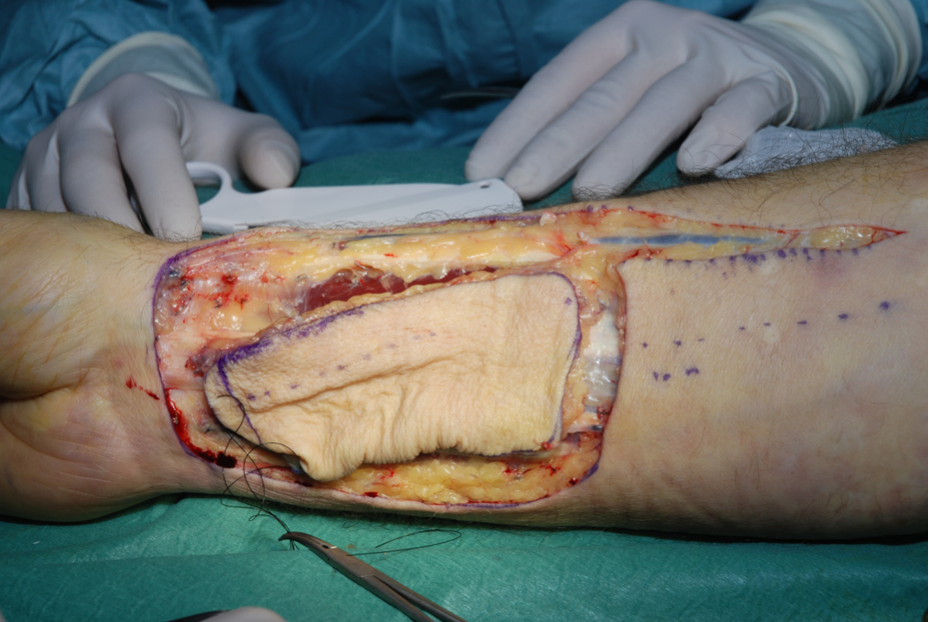
**Radial forearm flap for reconstruction of intraoral cavity**. Intraoperative view of the harvested flap, skin island

**Figure 2:**
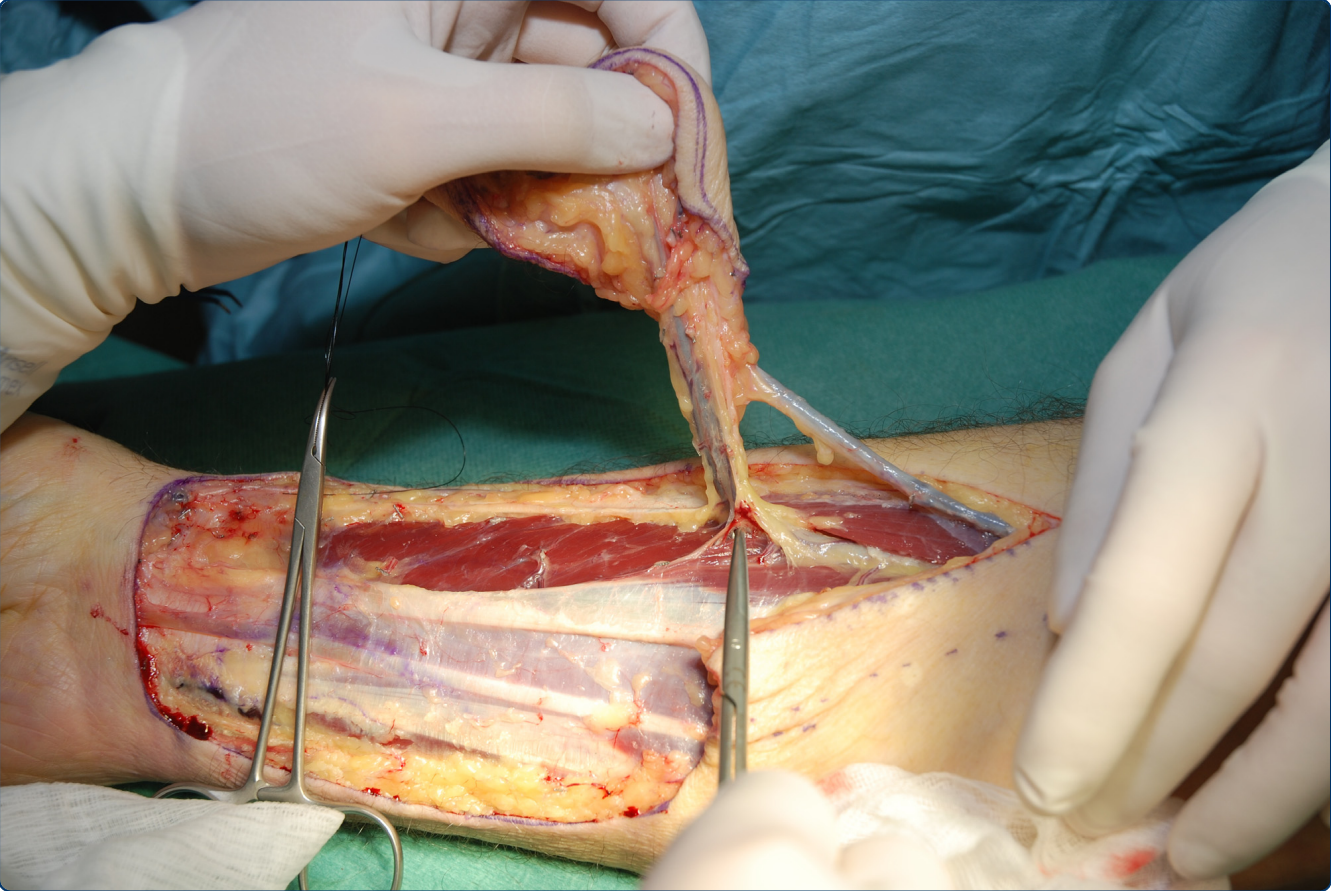
**Radial forearm flap for reconstruction of intraoral cavity**. Intraoperative view of the harvested flap, reverse view including the pedicle, fascia over the tendon is not removed to improve postoperative range of motion

**Figure 3:**
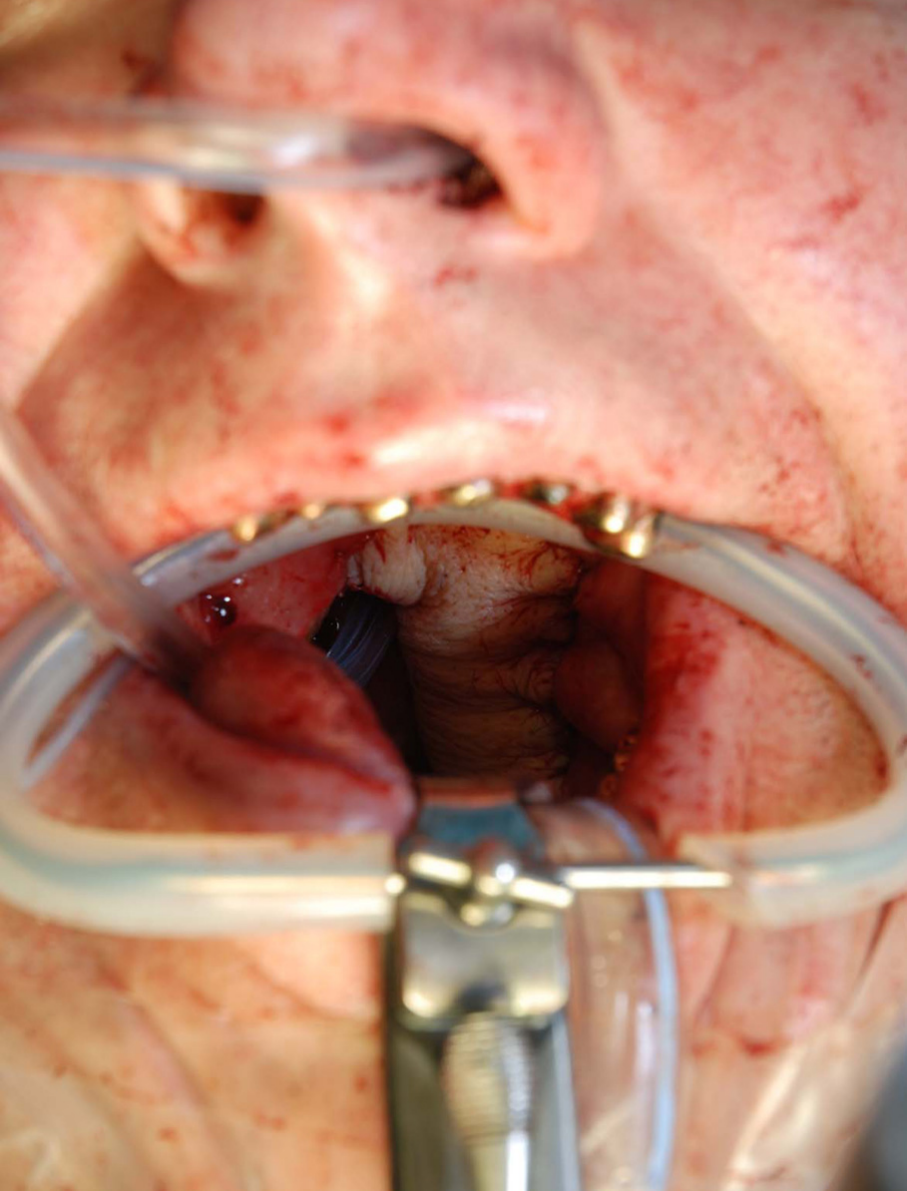
**Radial forearm flap for reconstruction of intraoral cavity**. Intraoperative view after flap inset in the oral cavity to cover a soft tissue defect

The radial forearm flap (Table 1) is the workhorse in intraoral and hypopharyngeal reconstruction. First described by Yang [9] this flap is based on the radial artery, shows a constant anatomy with a high-caliber and long pedicle. This flap is thin and provides a good pliability. Intraoperative view of the harvested flap, the skin island and the reverse view including the pedicle are demonstrated in figures 1 and 2 (the fascia over the tendon is not removed to improve postoperative range of motion). It covers defects in the oral cavity, as shown in figure 3, the tongue, trachea, oesophagus and the hypopharyngeal region. By including a radius bony segment an osteocutaneous flap can be raised to reconstruct small mandible defects [10]. Nevertheless the donor site shows aesthetic and functional problems: if primary closure is not possible, skin graft is necessary. Planning the radial forearm flap, the Allen test must be performed to ensure sufficient blood supply of the hand by the ulnar artery and palmar arch.

**Table 1:** Radial forearm flap

Flap size	Pedicle length	Pedicle diameter	Donor site morbidity	Patient's position	Special considerations
-Length: 12 cm (4-30 cm) -Width: 5 cm (4-15 cm) -Thickness: 1 cm -Bone: Length: 10 cm (6-14 cm), Width: 1 cm (0,7-1,5 cm)	-18 cm (15-22 cm)	-3 mm (2,5-3,5 mm)	-Cosmetic impairment -Diminishing blood supply of hand -Injury of superficial sensory branch of the radial nerve (neuroma)	-Back, arm on hand table, extended to 90 degrees	-Constant anatomy -Allen test

In patients with arteriosclerosis a reversed Allen test or an angiogram is advised.

### Flap raising

- Skin incision preserving fascia at the ulnar border of skin paddle

- Radial to flexor carpi radialis tendon: radial artery taken with fascia

- Dissection of artery and veins proximally; including subcutaneous veins

- Ligating distal artery and veins

- Primary skin closure or split thickness skin graft (in combination with collagen matrix)

The lateral arm flap (Table 2) was first described in 1982 [11]. The flap is perfused by the posterior radial collateral artery and it is placed at the lateral aspect of the upper arm. The flap can be raised with parts of muscle, tendon, humerus and sensory nerves. This flap is appropriate for facial defects because of its sleaziness and the possibility of reinnervation.

**Table 2:** Lateral arm flap

Flap size	Pedicle length	Pedicle diameter	Donor site morbidity	Patient's position	Special considerations
-Length: 12 cm (5-20 cm) -Width: 6 cm (3-12 cm) -Thickness: 1 cm (5-35 mm)	-6 cm (4-8 cm)	-1,5 mm (1-3 mm)	-Sensory deficit posterolateral arm -Lateral epicondylar pain -Scarring	-Back, arm on hand table, extended to 90 degrees	-Constant anatomy -If necessary skin closure by z-plasty or split thickness skin graft

### Flap raising

- Skin incision at the posterior circumference of the flap, taken with the underlaid fascia

- Dissection to the lateral intermuscular septum and including into the flap: posterior radial collateral artery

- Flap raising at the anterior margin of the flap sacrificing the posterior cutaneous nerve of the forearm and the radial nerve (loop)

- Dividing the intermuscular septum directly at the humerus

- Primary skin closure or split thickness skin graft

The anterior lateral thigh flap (ALT) (Table 3) was delineated by Song et al in 1984 [12]. The flap is perfused by a septocutanous perforator of the descending branch of the lateral circumflex femoral artery. The ALT can be used to reconstruct defects in nearly all areas in head and neck surgery. The flap's thickness depends on the amount of adipose tissue. It can be thinned and taken with a length of 25 cm and a width of 18 cm [13]. Wei published a failure rate of fewer than 2% [14]. The flap can be harvested with or without fascia. If fascia is taken, a functional loss can result.

**Table 3:** Anterior lateral thigh flap (ALT)

Flap size	Pedicle length	Pedicle diameter	Donor site morbidity	Patient's position	Special considerations
-Length: 16 cm (4-35 cm) -Width: 8 cm (4-25 cm) -Thickness: 2,5 mm (3-9 mm) -Muscle dimension: 2-25 cm	-12 cm (8-16 cm)	-2,1 mm (2-2,5 mm)	-Cosmetic impairment when closed by split thickness skin graft -functional loss due to fascia elevation	-Supine or lateral decubitus	-Inconstant perforator anatomy, meticulous preparation -when bulkiness is required-harvesting with vastus lateralis (or rectus femoris)

### Flap raising

- Preoperative hand held doppler examination half way between the ASIS and the lateral patella border to evaluate the perforating vessels

- Incision over the rectus femoris muscle 3 cm from the lateral intermuscular septum taken with fascia to include the intermuscular septum

- Identification of the pedicle, if necessary: muscular perforators (including vastus lateralis muscle), circumcision of the skin island

- Dissection of the vascular pedicle to the lateral circumflex femoral artery

The scapular flap (Table 4) was depicted by Dos Santos in 1980 [15]. The scapular flap takes its blood supply from the transverse septocutaneous branch of the circumflex scapular artery. The scapular flap should not touch the midline due to possible flap necrosis in the tip [16].

**Table 4:** Scapular flap

Flap size	Pedicle length	Pedicle diameter	Donor site morbidity	Patient's position	Special considerations
-Length: 18-20 cm (6-30 cm) -Width: 7-8 cm (4-16 cm) -Thickness: 2 cm (1,5-3 cm) -Bone: Length: 10-14 cm, Width: 2-3 cm, Thickness: 1,5-3 cm	-5-7 cm, extended up to 20 cm	-1,2 mm (0,8-1,4 mm)	-Cosmetic deformity if skin graft is necessary for closure	-Lateral decubitus position	-May provide non-hair-bearing skin

### Flap raising

- Medial incision with fascia overlying the infraspinatus muscle, blunt dissection of the flap from teres minor and infraspinatus muscle to the posterior muscle triangle: circumflex scapular artery

- Vessel loop around circumflex scapular artery and dissecting the pedicle preserving the bone perforators, if bone has to be harvested

- If necessary osteotomy of the scapula bone segment

The parascapular flap (Table 5) was described two years later by Nassif [17], it is nourished by the descending branch of the circumflex scapular artery. In 1986 osteocutaneous flaps including the lateral border of the scapula were described to reconstruct mandible [18]. The parascapular flap can yield up to 25 cm length and 10 cm width [19].

**Table 5:** Parascapular flap

Flap size	Pedicle length	Pedicle diameter	Donor site morbidity	Patient's position	Special considerations
-Length: 26 cm (6-32 cm) -Width: 12 cm (8-16 cm) -Thickness: 2 cm (1,5-3 cm) -Bone: Length: 12-14 cm, Width: 2-3 cm, Thickness: 1-2 cm	-5-7 cm, extended up to 20 cm	-1,2 mm (0,8-1,4 mm)	-Cosmetic deformity if skin graft is necessary for closure	-Lateral decubitus position	-May provide non-hair-bearing skin -Tissue pre-expansion by expander implantation is possible -Combination with the scapular flap

### Flap raising

- Flap elevation from distal to proximal preserving fascia

- Vascular pedicle appears immediately superior to the superior border of teres maior muscle, superior skin incision after exposure of the vessels, dissection proceeds from superior to inferior (vessels are observed at the inferior border of the teres minor muscle

- Continue dissection in the quadrangular space, ligature of branches to the lateral border of the scapula, teres minor muscle, dissection to the thoracodorsal artery

The latissimus dorsi flap (Table 6) can also be harvested as free flap for the coverage of extensive defects. The latissimus pedicle has an extra muscular length of 9 cm on average [20]. The latissimus can be used in large defects in head and neck. The thoracodorsal artery splits into two branches in the muscle: a horizontal and a vertical branch. Skin islands can be designed following the branches, so the indication for the flap can be enlarged for perforating defects of the oral cavity or the neck area [21]. Even an osteocutaneous transfer including a rib is described [22].

**Table 6:** Latissimus dorsi flap

Flap size	Pedicle length	Pedicle diameter	Donor site morbidity	Patient's position	Special considerations
-Muscle: Length:35 cm (21-42 cm) -Width: 20 cm (14-26 cm) -Thickness: 1,5 cm (0,5-4,5 cm) -Skin: Length: 18-35 cm, Width: 7-20 cm, Thickness: 2,5 cm (1-5 cm)	-8,5 cm (6,5-12 cm)	-3 mm (2-4 mm)	-Cosmetic deformity if skin graft is necessary for closure -Weakness of shoulder possible -High rate of seroma	-Lateral decubitus position -Prone or supine with 45° lateral tilt	-Skin alone can be harvested as perforator flap (TAP flap)

### Flap raising

- Incision along the mid axillary line

- Identification of the anterior border of the latissimus muscle

- Branch of thoracodorsal artery to the serratus anterior muscle leads to the vascular pedicle of the latissimus muscle

- Circumcision of the skin paddle (fixation of skin paddle to the muscle with stay sutures), elevation of the muscle creating a muscle strip between the cranial pole of the skin paddle and the vascular pedicle

- Dividing side branches of the thoracodorsal vessels (use for the TAP flap without any muscle)

The transverse rectus abdominis flap (TRAM) (Table 7) was first described by Holmström in 1979 for breast reconstruction [23]. The deep inferior epigastric perforator artery flap (DIEP) (Table 7) came up in 1989 [24]. Both flaps are based on the inferior epigastric artery, which presents constant anatomy with a long pedicle of up to 10 cm. Advantage of the DIEP flaps in comparison to the TRAM flap is the reduction of abdominal weakness or herniation [25].

**Table 7:** TRAM/DIEP flap

Flap size	Pedicle length	Pedicle diameter	Donor site morbidity	Patient's position	Special considerations
Muscle: -Length: 25 cm (23-29 cm) -Width: 6 cm (4-8 cm) -Thickness: 1,5 cm (0,7-2 cm) Skin: -Length: 13 cm (10-20 cm) -Width: 25 cm (20-20 cm) -Thickness: 2,5 cm (1-6 cm)	-7 cm (6-8 cm)	-3,5 mm (3-5 mm)	-Herniation	-Supine position	-Supercharging with superficial epigastric artery or vein -Mesh implantation to restore abdominal strength at donor site

The rectus abdominis muscle can also be taken on the same pedicle with or without a skin island to fill up dead space after soft tissue or bone removal, and if necessary covered with skin graft. If the skin is orientated vertically, the flap is named VRAM flap (vertical rectus abdominis musculocutaneous flap).

### Flap raising

- Skin incision in the lower abdominal wall, dissection of the subcutaneous fat preserving superficial inferior epigastric artery and vein (in case of performing a SIEA flap), cranial skin incision

- Dissection from the lateral to the medial aspect taken care of the perforators in the lateral and medial line on both sides

- Pick the best perforator, temporarily clip out the other perforators and watch the flap's circulation

- Selection of the best perforator (sometimes 2-4 perforators are necessary), incision of the fascia longitudinally in direction of the pubis, intramuscular dissection of the perforator, save segmental intercostals nerves, rectus muscle can be taken with the perforators (TRAM)

- Deep inferior epigastric artery is taken as long as necessary for the pedicle length

- After flap transplantation repair of the anterior sheath of the rectus with a braided resorbable suture (mesh at big fascia defects which cannot be closed without tension), adherence zone undermining up to the xiphoid process, closure of abdomen in layers after exteriorizing the umbilicus

**Figure 4:**
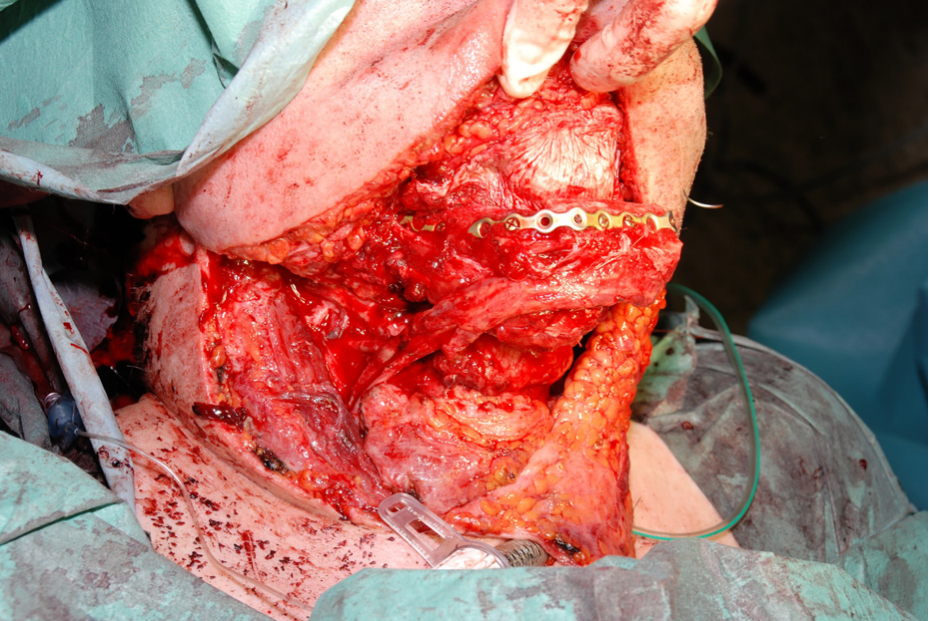
**Fibula osteocutaneous flap for reconstruction of the mandible**. Intraoperative view of the inset fibula after manbible resection due to cancer (50 years old male)

**Figure 5:**
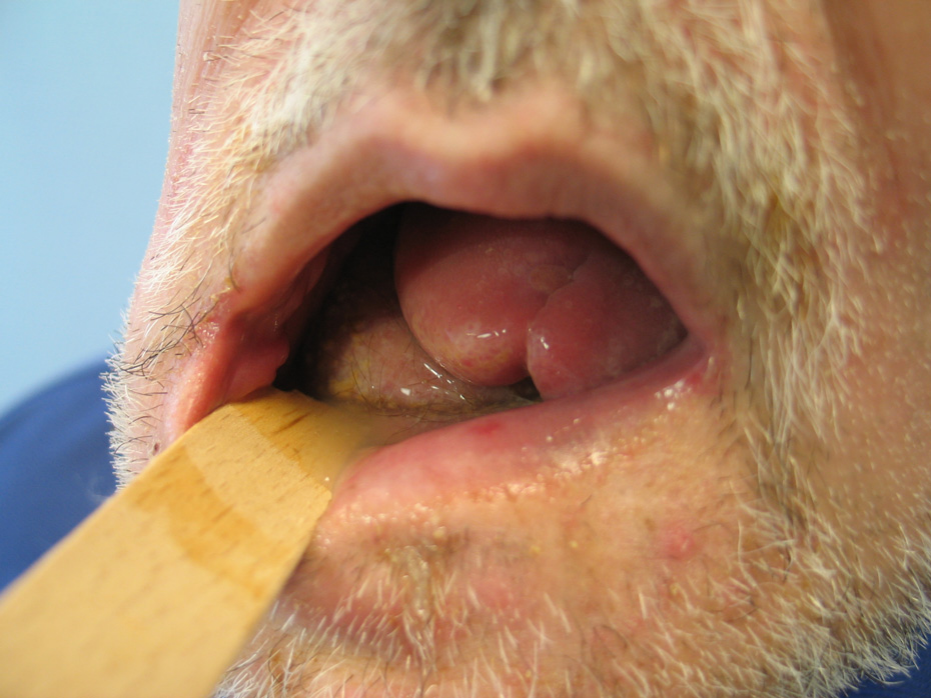
**Fibula osteocutaneous flap for reconstruction of the mandible**. Postoperative view of the intraoral cavity, note the well circulated skin island

**Figure 6:**
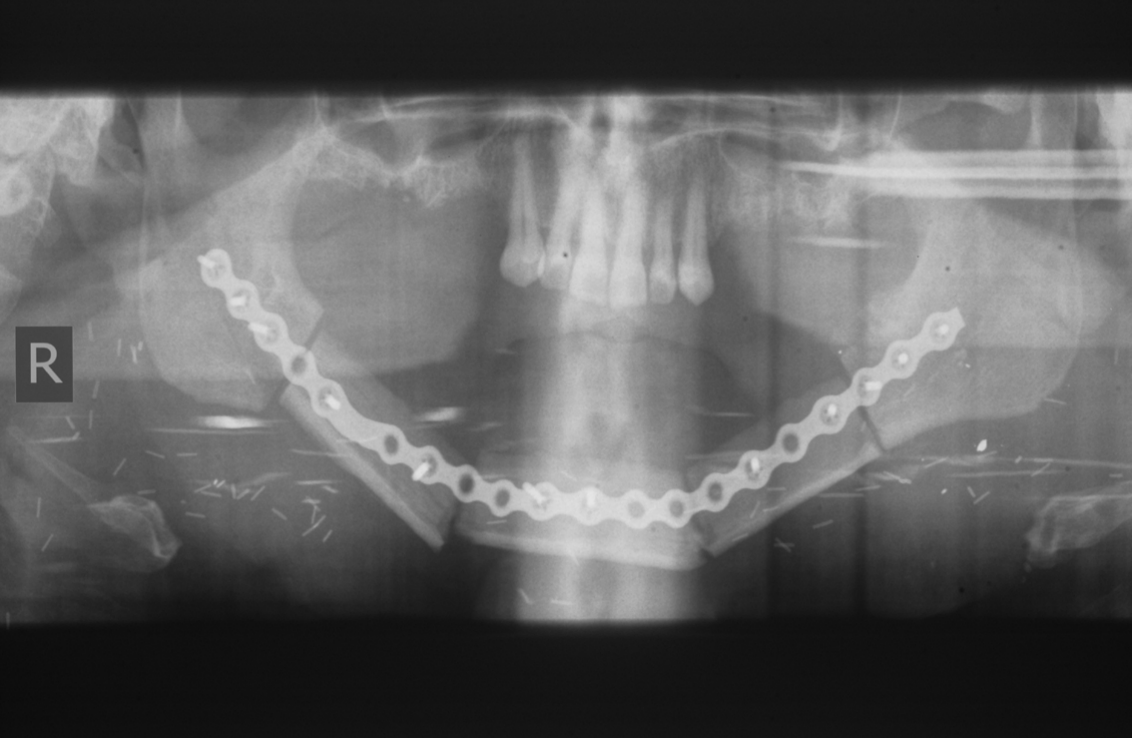
**Fibula osteocutaneous flap for reconstruction of the mandible**. Postoperative X-ray 3 months after transplantation

**Figure 7:**
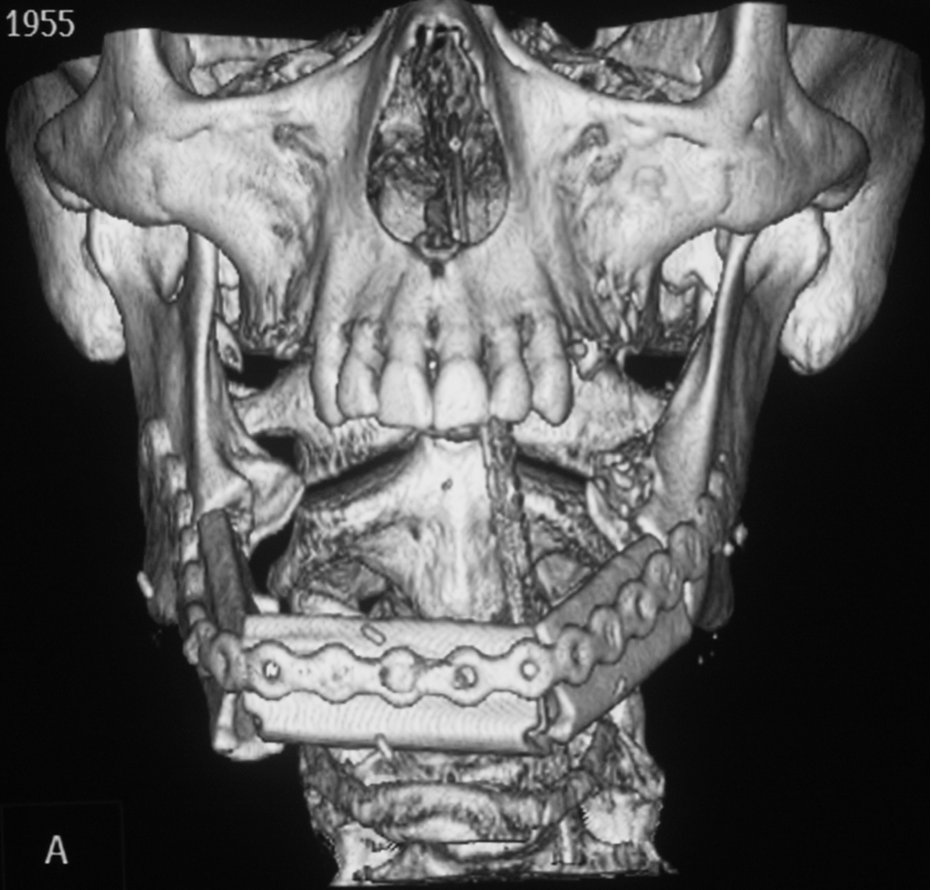
**Fibula osteocutaneous flap for reconstruction of the mandible**. CT reconstruction of the mandible 4 month after transplantation

In 1989 Hildago performed the first fibula osteocutaneous flap (Table 8) for reconstruction of the mandible [26]. This flap allows to reconstruct defects exceeding the length of 75% mandible as the fibula is the longest bone flap with up to 25 cm [27]. An intraoperative situs with the inset fibula after mandible resection due to cancer is shown in figure 4. Figure 5 shows the postoperative intraoral mucosa. The same patient is shown in the x-ray 3 months after operation and in the CT 4 months after transplantation in figures 6 and 7. It is necessary to save the proximal and distal end to avoid instability of ankle and knee. Meticulous dissection preserves the peroneal nerve. The vascular pedicle is the peroneal artery. This flap can be harvested with one or two skin paddles, based on perforators from the peroneal artery, which travel through the soleus muscle or the posterior intermuscular septum [28]. The donor site often requires a split-thickness skin graft.

**Table 8:** Fibula osteocutaneous flap

Flap size	Pedicle length	Pedicle diameter	Donor site morbidity	Patient's position	Special considerations
Skin: -Length: 12 cm (10-32 cm) -Width: 6 cm (4-14 cm) Muscle: (lateral hemi-soleus): -Length: 16 cm (18-30 cm) -Width: 8 cm (6-15 cm) Bone: -Length: 16 cm (6-26 cm) -Thickness: 2 cm (1-3 cm)	- Up to 10 cm, depending on bone length and location of donor site (proximal or distal)	-1,5 mm (1-2,5 mm)	-Cosmetic deformity -Limitations and discomfort in ankle function -Peroneal nerve palsy	-Supine position, knee flexed 90°, pelvic girdle internally rotated -Prone position	-Preoperative vascular study could be useful (angiogram)

### Flap raising

- Skin incision over peroneus muscle keeping 2 cm to the posterior intermuscular septum

- Visualizing of the perforators

- Dissection of the posterior intermuscular septum, soleus muscle and flexor hallucis longus muscle proximal to the skin paddle, identification of peroneal vessels (vessel loop)

- Circumcision of the skin paddle, elevation bone, skin paddle and posterior intermuscular septum

- Distal osteotomy of fibula (6-8 cm distance to the ankle), proximal osteotomy of fibula (5 cm distance to fibula head)

- Anterior septum incised between the osteotomies, periosteum remains untouched; harvesting of bone and distal ligation of fibular artery and vein after dislocation of fibula and incising interosseous membrane

The free jejunal graft (Table 9) was the first free flap reported in humans in 1959 by Seidenberg [29]. The jejunal flap is used to reconstruct defects in oesophagus and after pharyngectomy. The flap is based on the jejunal artery sourced by the superior mesenteric artery. The pedicles length is 4-6 cm with a diameter of 1,5-2,5 mm. The jejunum length for transplantation ranges from 7 to 25 cm. The jejunal flap shows fairly high complication rate [30] as ileus or peritonitis. The jejunal flap is very susceptible to harmful effects of ischemia (maximum 2 hours).

**Table 9:** Free jejunal graft

Flap size	Pedicle length	Pedicle diameter	Donor site morbidity	Patient's position	Special considerations
-Length: 7-25 cm -Bowel lumen diameter: 3-5 cm	-5 cm (4-6 cm)	-2 mm (1,5-2,5 mm)	-Intestinal leak or stricture -Peritonitis -Ileus -Adhesive bowel obstruction	-Supine position	-Decrease ischemia time -Mal odeur and secretion

### Flap raising

- Midline laparotomy, ligament of Treitz is identified in the left upper quadrant, small bowel is followed 40-60 cm below ligament of Treitz

- Vascular arcades identified by transillumination, jejunal segment longer than the defect has to be selected, marking the segment's proximal portion

- Complete division of mesentery, vasa recta are clamped

- Bowel clamping with atraumatic clamps, isolation of the jejunal segment, harvesting of the bowel using intestinal stapling

- Free dissection of the jejunal artery and vein

Sanders described the first iliac crest flap (Table 10) using the deep circumflex iliac artery as a pedicle [31]. This flap matches to restore defects of half a mandible [29]. It allows insertion of enosseous dental implants. The iliac crest flap offers an enormous amount of bone, so this flap seems to be predestinated for reconstruction of mandible. This flap can be harvested with an associated skin and/or muscle paddle (transverses abdominis, internal and external oblique abdominis muscle) for reconstruction of composite defects. The donor site may result in hernia, which can be avoided by careful closure techniques.

**Table 10:** Iliac crest flap

Flap size	Pedicle length	Pedicle diameter	Donor site morbidity	Patient's position	Special considerations
Skin: -Length: 15 cm (10-20)cm -Width: 10 cm (5-15 cm) Bone: -Lenght: 4 cm (3-8 cm) -Width: 2 cm (1-3 cm)	-2 cm (1,5-3 cm)	-1,5 mm (0,8-2 mm)	-Subcutaneous seroma -Sensory loss lateral thigh region -Lymphedema of the lower extremity -Abdominal wall hernia	-Supine position	

### Flap raising

- Incision 2 cm superior to the connection of pubic tubercle and anterior superior iliac spine, starting lateral from femoral artery

- Ligature of the superficial epigastric vessels

- Incision of the inguinal ligament parallel to its fibers, transsection of the internal oblique muscle, deep circumflex iliac artery is palpated in the groove formed by transverses and iliacus muscle, blunt dissection (vessel loop)

- Transsection of the muscles from the iliac crest

- Distal osteotomy of the iliac crest, ligation of the vascular pedicle at the distal osteotomy, proximal osteotomy, flap harvesting

## Discussion

Every particular area requires special qualities and tissues to restore function by flap transplantation. Choosing the most appropriate flap implies the proper diagnosis of defect size and its complexity, as well as necessary tissue components and the patient's condition.

### 1. Soft tissue defects

The radial forearm flap requires the integrity and patency of the palmar arch. Disadvantages are possible cold intolerance and an unsightly scar at the donor site with limitation in motion. The large diameter of the vessels and the long pedicle are very helpful when planning and performing the anastomoses. The radial forearm flap can be harvested in parallel to tumor resection which helps to keep operation time low. To minimize problems with skin graft healing due to underlying tendons it is suggested to harvest the flap without fascia and to use a collagen matrix (such as Matriderm^®^) underneath the skin graft. In this way the aesthetic outcome and the range of motion are improved.

The lateral arm flap can be used the restore intraoral defects. Koapting the posterior cutaneous nerve with the lingual nerve was described to feature a high success rate of neurocutaneous reinnervation [32]. In comparison to the radial forearm flap the harvesting of the upper arm flap is more demanding because of the vessel's smaller diameter, the deeper location of the pedicle and the close relationship to the radial nerve. The subcutaneous fat layer is thinner in the radial forearm flap, especially in adipose patients [33]. The average length of the pedicle is 7 cm [34]. The pedicle of the radial forearm flap is described with up to 47 cm [35]. In case of reconstruction after radical neck dissection a longer pedicle is often necessary. The lateral arm flap mostly cannot be harvested simultaneously to the tumor resection because of the nearby localisation to the head and neck area.

**Figure 8:**
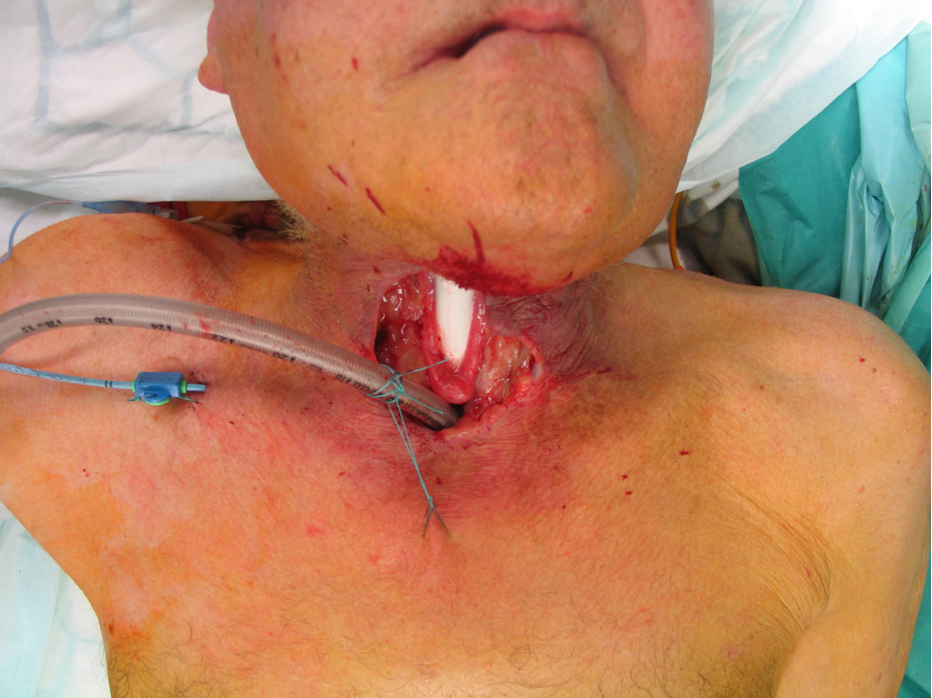
**Anterior lateral thigh flap for hypopharyngeal reconstruction (64 years old male with laryngeal and hypopharyngeal cancer)**. Preoperative view of the hypopharyngeal defect, note: two linings are required to cover the hypopharyngeal and the skin defect

**Figure 9:**
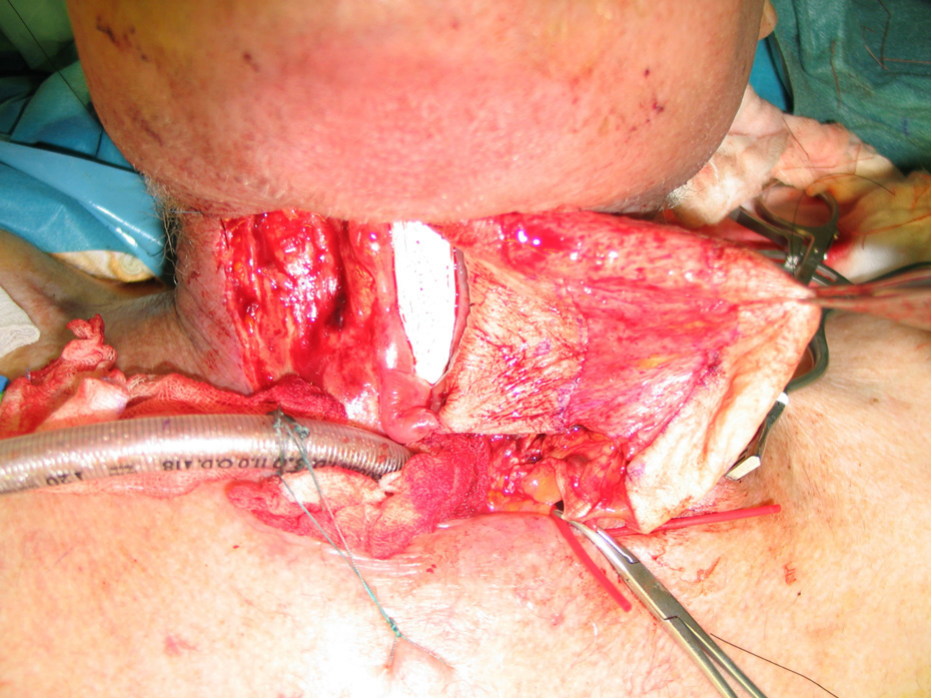
**Anterior lateral thigh flap for hypopharyngeal reconstruction (same patient)**. Intraoperative view of the inset flap

**Figure 10:**
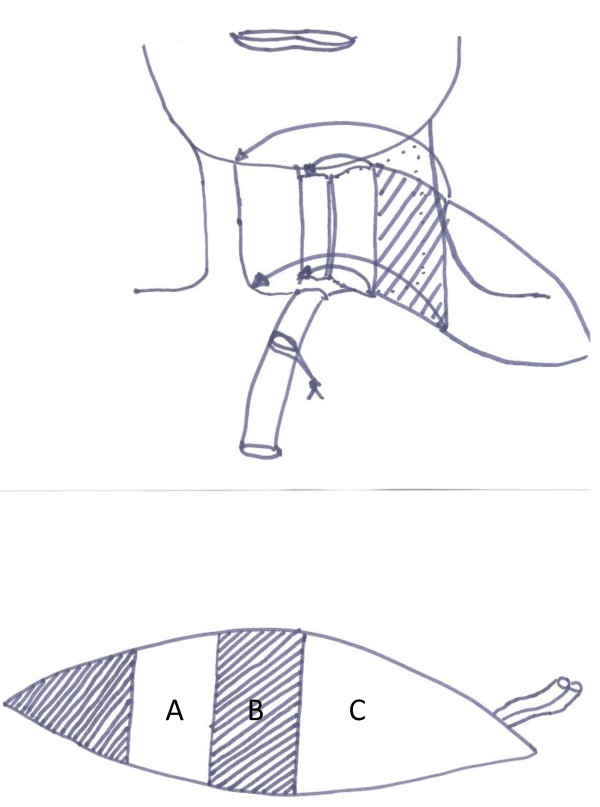
**Anterior lateral thigh flap for hypopharyngeal reconstruction (same patient)**. Schema of deepithelialisation to reconstruct both linings (as shown in figure 9), shaded area is deepithelized; A is used to built the tube, B is used to cover the tube, C is used to cover the defect (external skin)

**Figure 11:**
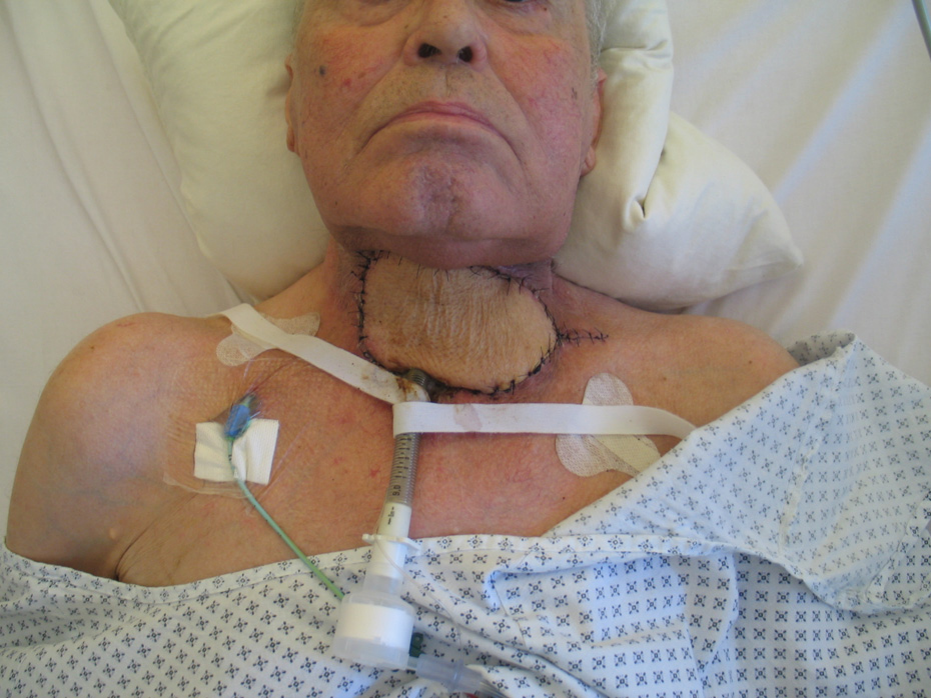
**Anterior lateral thigh flap for hypopharyngeal reconstruction (same patient)**. Postoperative external view

The ALT flap shows difficulties in obese patients because of the thickness of the subcutaneous fat tissue. In these cases it can be derogatory to cover defects in the oral cavity and the hypopharyngeal region due to its thickness. The course of the pedicle shows enormous variation: the pedicle can run through the vastus lateralis instead of going through the intermuscular septum [36]. One advantage of the ALT over the radial forearm flap is the donor site closure: most of donor sites of ALT can be closed primarily, skin graft was only necessary in 2 of 39 flaps for restorage of the upper aerodigestive tract [37]. Donor site can be closed primarily when the width does not exceed approx. 8 cm. Even when harvesting a large portion of the vastus lateralis muscle there is no significant functional or aesthetic impairment [38]. The ALT can be tubulized to reconstruct hypopharyngeal structures. Depending on the different perforators (up to 3 or 4) the ALT can be designed to cover defects with two linings e.g. intraoral or extraoral by dividing the skin paddles based on the respective perforator as shown in figures 8, 9, 10 and 11.

The latissimus dorsi flap shows constant anatomy with a reliable pedicle and an enormous length and width. A simultaneous harvesting of the flap to head and neck cancer surgery is nearly impossible, which prolongs operation time and demands intraoperative position change of the patient. The donor site morbidity of the latissimus dorsi is quite high: seroma can occur, and shoulder function can be impaired [39]. The latissimus dorsi muscle is mostly too bulky for reconstruction in the intraoral cavity because of the subcutaneous fat layer. To avoid bulkiness using the same donor site the perforator version of this flap (TAP flap) is recommended.

As in the latissimus dorsi flap scapular/parascapular flaps cannot be raised at the same time as tumor ablation. The donor site of the scapular/parascapular flap has fewer complications compared to the latissimus donor site, as it creates no hole.

TRAM/DIEP and isolated rectus abdominis muscle flaps can be harvested parallel to tumor resection, no intraoperative patient change is necessary. These flaps show a save and certain blood supply with a long pedicle. The thickness of these flaps depends on the patient's general size. If possible, DIEP flap should be chosen rather than TRAM flap to avoid abdominal wall weakness and herniation [24].

Muscle flaps provide good tissue bulk, but in the long run they begin to shrink and sag, which makes long-term results inconstant to the postoperative situation.

The free jejunal graft and its complications were described by Coleman [30]. Despite the advantage of the tubed nature of the jejunum and its natural lubrication there are many disadvantages: Coleman described a failure rate of 13,5% with a mortality of 5%. Pharyngo-cutaneous fistula occurred in 33 of 101 patients. 18 of 101 patients suffered from stricture or stenosis at the bowel anastomoses, 4 patients suffered from abdominal wall dehiscences. To harvest the flap, laparotomy or laparoscopy-assisted approaches to the abdomen are required which carry their own risk. Besides the jejunal flap shows very low tolerance to ischemia. To minimize the risks and complications we try to avoid free jejunal graft, because the donor site morbidities of radial forearm flap or ALT flap diminishes to those of the free jejunal graft. A tube with a sufficient diameter (up to 3 cm in ALT flap) can be formed by most of the fasciocutaneous flaps.

### 2. Bony defects

For restoration of bony defects the fibula osteocutaneous flap is convenient because of the possibility to raise the flap parallel to tumor resection in a two team approach. The quality of the skin paddle is comparable to the radial forearm flap but the hairiness of the skin paddle can incommode the patient, especially in intraoral situations. The vascular pedicle can reach up to 15 cm with a large diameter which allows performing anastomoses easily [40]. The high frequency of arteriosclerosis in the lower leg vessels especially in these patients suffering of oral cancer must be included in the flap choice. We recommend to perform an angiography before planning a fibula flap to uncover arteriosclerosis or the absence of the peroneal artery. The donor site morbidity of the fibula flap shows hypoesthesia at the lateral malleolus, pain and oedema and a reduction of the motor range of ankle when the superficial peroneal nerve is injured [1]. In special cases it can become necessary to use an iliac crest flap for bone reconstruction (if possible with the defect's size).

The iliac crest flap has a reliable pedicle and offers a wide variation of combinations of bone, muscle and skin flap. This flap is highly suited to reconstruct mandible. The skin paddle is often bulky; especially in obese patients it is difficult to harvest this flap. If the flap is too bulky for the recipient site it can be harvested as crest bone with oblique abdominis muscle and a skin graft without a skin paddle. In many times herniation occurs, even long lasting pain, neuropathy, gait disturbance and impotence were described [41]. The natural contour of the iliac crest is ideal to reconstruct lateral mandibular defects, by contrast to the free fibular flap osteotomies are rarely needed in smaller defects. This flap can be harvested parallel to tumor resection, which spares operating time. Alternatively, especially for smaller defects a radial forearm flap with a part of radial bone can be performed.

### 3. Flap choice

Disa found that most defects can be reconstructed with jejunum, rectus, forearm and fibula flap [42]. Neligan postulated that 2 flaps are sufficient for most intraoral reconstructions: radial forearm flap for restoration of the intraoral lining [34], and the fibula osteocutaneous flap for mandible reconstruction, as it provides a good quality and quantity bone stock with a reliable skin paddle [43].

Reconstruction of hypopharynx is a challenge for the plastic surgeon. A watertight conduit is needed, that can be punctated tracheoesophageal for speech. This is rarely successful in free jejunal transfers due to the mucus that leads to a wet-sounded voice and a loss of stability [44].

Depending on defects size, required pedicle length and individual donor site situation we recommend reconstruction of soft tissue in oral cavity and hypopharynx with fasciocutaneous tissue from radial forearm, lateral arm, anterior lateral thigh or parascapular/scapular (Table 11). Due to the intraoperative position change of the patient the parascapular flap in head and neck coverage is not preferred. In most cases the ALT flap offers the same flap criteria. The decision whether a free flap or a pedicled flap should be performed is not based on cost or morbidity, as there are no significant differences between free and pedicled flaps in cost and morbidity, except the time in the operation room [44].

**Table 11:** Defect algorithm Oral cavity

Soft tissue defect	
Floor of mouth	- Radial forearm flap
	- Lateral arm flap
	- TAP
	- Alt

Tongue	- ALT
	- Radial forearm flap
	- Lateral arm flap
	- TAP

Palate	- Radial forearm flap
	- Lateral arm flap
	- TAP
	- ALT

Bone Defect	

Mandible/Palate	- Fibula osteocutaneous flap (up to 25 cm bone)
	- Iliac crest flap (14-16 cm bone)
	- Radial forearm osteocutaneous flap (30% radius circumference)
	- Scapular osteocutaneous flap (up to 10 cm bone)

Hypopharynx	

Partial defect	- Radial forearm flap
	- Lateral arm Flap
	- TAP
	- ALT (strongly recommended when external skin coverage is necessary- wrap flap)
	- Latissimus dorsi muscle

Circumferential defect	- ALT (wrap to build a tube)
	- Jejunum
	- Latissimus dorsi muscle
	- TRAM/DIEP

Defects creating dead space	- Rectus abdominis muscle
	- ALT with vastus lateralis

In our experience procedures should be performed in a single stage procedure to reduce hospitalisation and complications which in many cases can be attributed to secondary wound infections, radiation depending problems and strictures due to secondary healing. ENT surgeons and Plastic surgeons have to establish an attentive multi disciplinary approach to provide optimal oncologic and reconstructive procedures for their patients.

## Consent

Written informed consent was obtained from the patients for publication the accompanying images in this review. A copy of the written consent is available for review by the Editor-in-Chief of this journal.

## Competing interests

The authors declare that they have no competing interests.

## Authors' contributions

ICW and HF worked on the literature research and assembled the article together. All authors read and approved the final manuscript.
